# Observations on the Treatment of Some Forms of Obstruction of the Bowels

**Published:** 1853-04

**Authors:** 


*Observations on the Treatment of some forms of Obstruction of the Bowels.* By Thomas Aickin, M. D.

The fatal termination of the affection which has been termed "Ileus," or the "Iliac Passions," seems to be owing in many instances to the prostrating influence of the symptoms usually attendant on the local visceral lesion, independent of any progressive change of a necessarily fatal character occurring in the latter. Abercrombie's statement, that "ileus may be fatal without obstruction," and that "it does not appear to be necessarily connected with obstruction in any part of the canal," is calculated to excite some doubt as to the propriety of directing treatment almost exclusively to the removal of an obstruction, the nature, and possibly the existence of which continue to be matter of surmise and not of positive diagnosis.

I have had opportunity of observing the progress of some cases of obstinate constipation, in which the symptoms of ileus set in with great intensity. I have in some instances found it possible to subdue those symptoms many days before the removal of their assumed cause and the reappearance of feculent discharges; and I have remarked that the prolonged retention of the latter was not productive of dangerous consequences when the abdominal pain, vomiting, and general distress, were in the first instance controlled by appropriate treatment.

The first case which served to convince me of the injurious effects resulting from the repeated administration of purgatives, to the exclusion of other remedies, happened to be one of those in which purgatives seemed to be specially indicated. The patient, a young lady, had been subject to torpid bowels and pain, chiefly in the cœcal region; vomiting and severe abdominal pain supervened on protracted constipation. She was harassed for three days with these symptoms; the bowels remaining obstinately confined, notwithstanding the repeated exhibition of purgative medicine both by the mouth and per rectum; the stomach retained nothing, she had no sleep, and scarcely an interval of ease, up to the morning of the fourth day from the invasion of the acute symptoms, when I saw her for the first time. I found her much exhausted, the pulse very small, the extremities cold, and the nausea and straining to vomit most distressing; the bowels had not been moved for many days. On examination, a large and resistant mass was plainly perceptible in the cœcal region; it was about the size of a large orange; there was some surrounding tenderness; a blister had been applied over this region, and large quantities of purgative medicine administered before I saw her. Finding that the treatment previously adopted had been ineffectual, and that her strength was rapidly failing, I at once put her on calomel and opium—two grains of the former and one grain of the latter, in pill, were given, warmth applied to the extremities, hot stupes to the abdomen, and abstinence from all fluids, except cold water in small quantities, enjoined; the pill was retained, and the stomach became quiet. The calomel and opium were repeated every fourth hour. On the following morning—namely, the fifth day of her illness, I found that she had obtained upward of six hours of sound and refreshing sleep, the vomiting had been arrested, but the abdominal tenderness had increased. General reaction was now fully established; the pulse, which on the preceding day was almost imperceptible, had become full and strong, and the countenance flushed. Twelve ounces of blood were taken from the arm, and in a few hours after leeches were placed over the cœcal region, and followed by a large poultice. The calomel and opium were persisted in. On the sixth and following days, she was almost free from general or local suffering; the gums became tender. Croton oil was now given in drop doses, which caused some return of the tormina; a blister was applied over the cœcal tumor; the blistered surface was dressed with mercurial ointment. On the tenth day, there were some small fragments of hardened fæces discharged after the administration of an enema; on the following days, gentle frictions with mercurial ointment were repeatedly made over the mass in the cœcum, which ultimately broke up and was discharged piecemeal. Its fragments were of a dark color, and strikingly resembled shoemaker's wax blended with some fibrous matter, both in their color and amazing tenacity. This patient was sustained by the administration of enemata of beef-tea, thrown up by the tube. Three pints were thus given every day from the subsidence of the acute symptoms, until the stomach became able to tolerate food. Her recovery was perfect.

In another case of obstruction, accompanied in its early stage by the usual distressing symptoms, the sensation of pain and distress was chiefly referred to the umbilical region, several attempts had been made to open the bowels by the administration of oily purgatives, but without effect; the incessant retching proved so distressing that in the course of four or five hours from its commencement the patient became much exhausted. Calomel and opium were administered in pills, repeated every fourth hour. Leeches were applied over the umbilical region, and iced water was allowed in small quantities. Under this treatment the vomiting became less troublesome, and the abdominal pain and tenderness were much diminished; large linseed-meal poultices were applied to the abdomen, and afforded much relief. Eight days elapsed from the commencement of the treatment of this case before fæcal evacuations were procured; no distressing symptoms were manifested after she was brought under the influence of opium. On the fourth day, the gums became tender; the calomel and opium were now given at longer intervals. Attempts were again made to free the bowels by repeated doses of castor oil, and by large enemata, but up to the eighth day without effect; on and after which liquid fæces were discharged. Blistering the abdomen and mercurial dressings were also in this case attended with manifest advantage. Her recovery was unchecked by any untoward symptoms.

In another case of obstinate constipation, occurring in a lady who had been constantly obliged to resort to purgatives, the symptoms of obstruction set in with great violence. I found her on my first visit in a state of great exhaustion; she was straining at vomiting, and brought up frothy mucus in large quantity. She was so much sunken as to be unable to reply to the queries put to her; the pulse were scarcely perceptible, and the extremities cold. As soon as the cessation of the retching allowed a momentary pause, I gave her four drops of black-drop in a teaspoonful of water, had warmth applied to the extremities, and enjoined as perfect repose as the nature of her suffering would admit of. After the lapse of an hour, I found that the opiate had quieted the stomach, and that reaction was beginning to set in. Hot turpentine stupes were applied to the abdomen, and ten grains of calomel and a drop of croton administered. Finding, after the lapse of some hours, that pain followed the purgative, and that the vomiting returned, I repeated the opiate, and directed the administration of a large enema containing turpentine and oil. The bowels continued obstinate, and the paroxysms of pain, which were evidently owing to the stimulus of the purgative, became very distressing. Black-drop was again given; she passed a bad night, occasionally straining very much at vomiting, and brought up dark-brownish matter in small quantities. On the day following, finding that the bowels had resisted the purgative, and the abdomen continuing very much swollen and tender, I prescribed pills of calomel and opium; these were regularly persisted in for upward of three days, until the gums became tender. After she had taken three or four pills, the symptoms were much improved; large enemata were occasionally exhibited, and her strength was supported by fluid nourishment given in small quantities. About the tenth day, the bowels were slightly moved, and on the two following days dark-colored fœtid discharges were freely passed. It may be remarked, concerning this case, that the first decided relief was obtained by opium, while the removal of the obstruction, whatever its immediate cause may have been, was obviously aided by the mercurial action, inasmuch as purgatives produced no effect previous to the manifestation of the latter; a few doses of castor oil were then sufficient to procure stools, after which nothing occurred to interfere with her recovery.

The causes of ileus are various, and their differential diagnosis is, in many cases, impossible. The affection may occur, as noticed by Abercrombie, in "the simple form, without any previous disease," or "with previous disease which deranges the muscular power without mechanical obstruction," or it may occur "with mechanical obstruction." Now, it is sufficiently obvious, that when distressing or dangerous spmptoms arise as consequences of an undiscovered or merely surmised lesion, our treatment should in the first instance be directed to their alleviation or removal. The possibility of effecting this, even when their exciting cause cannot be directly interfered with, is illustrated by everyday practice. Thus, the symptoms following obstruction of the bowels which are developed in virtue of an exalted reflex action, are controlled by sedatives which diminish the excitability of the nervous centers, and render them less capable of propagating morbid impressions originating in local disturbance of either function or structure. Hence the necessity of resorting to this class of remedies in order to check the diffusion of excited or perverted action, arising from any cause whatsoever. Hence the necessity of exhibiting opium in ileus occurring in any form, "with or without mechanical obstruction," when once the effects of an over-excited or perverted innervation are strongly developed. According to this view, it is applicable to every form of the affection accompanied by urgent symptoms, whether arising from intussusception or strangulation, from peritoneal adhesions or bands of lymph, internal or external irreducible hernia, impacted fæces, concretions, stricture, muscolo-enteritis, and simple distension, with loss of power, &c., &c. Abercrombie states, that "a modification of the disease yields to a full opiate more readily than to any other mode of treatment." Mackintosh observes, that in ileus "opium seems to increase rather than diminish the laxative effects of medicines." The profound sagacity of Sydenham enabled him to discover a mode of treatment scarcely improved on by subsequent practice. For the treatment of the iliac passion, he directs that "blood be taken from the arm; after a few hours, twelve grains of scammony and a scruple of calomel are to be given; if rejected by vomiting, twenty-five drops of the liquid laudanum are given in half an ounce of strong cinnamon water. Should the vomiting cease, the purge is to be repeated; but should it return along with pain, and the cathartic be retained, the same dose of laudanum is to be again given, and to be repeated every fourth or sixth hour until the motion of the intestine be perfectly quieted; after the action of the purge, the opiate is to be repeated twice, thrice, or oftener, during the day, until the pain and vomiting have wholly ceased." Had Sydenham been aware of the remarkable effects of mercury pushed to salivation when it failed to purge in such cases, there would have been nothing to add to this method of treatment.

Amongst the many remedies employed in this affection, tobacco and belladonna enemata have been occasionally successful; the former is a remedy of much power, and its depressing effects are well known; the latter has been extolled by some modern practitioners, but is confessedly a dangerous remedy. Hanius, Wagner, and some others, have seen it followed by good results; the former has given the infusion of the root of belladonna, made by infusing a dram in as much boiling water as would yield one ounce on straining; this, with an equal quantity of infusion of chamomile, is administered as an enema. (Canstat, *Med. Clin.*) Messrs. Trosseau and Reveil direct dried belladonna leaves (gr. xv, to ℥ss.) to be infused in eight ounces of boiling water for one hour, and strained; this quantity is to be administered as a glyster in cases of ileus from strangulated hernia. Amongst other remedies occasionally successful, are enemata of large quantities of warm or cold water, thrown up by O'Beirne's tube. Dr. R. H. Townsend, of Philadelphia, succeeded in overcoming an obstruction which threatened speedy death, by first throwing a quart of ice-cold water into the rectum, then suspending the patient by his feet to the ceiling of the chamber, and kneading the abdomen with considerable force; the signs of obstruction immediately ceased, and in fifteen minutes the injected water with fæculent matter were evacuated. (Wood's Practice of Physic.)

I have succeeded in freeing the bowels in a case which had resisted powerful purgatives for a week by pumping up large quantities of strong soap-suds, by means of the tube and syringe. Rokitansky thinks that injections of air are applicable to cases of invagination. To be of any use, however, they must be employed before adhesions take place between the opposed surfaces of the invaginated bowel.

A host of remedies, familiar to most persons, have been from time to time resorted to for the relief of ileus; some of them are undoubtedly productive of dangerous effects. I have known shot to be swallowed, and strange to say the relief was immediate; but such remedies are rarely successful. In every case, it is indispensable that a minute examination of the abdomen should precede the use of remedies. There are instances recorded of death from strangulated hernia unnoticed during life; stricture of the rectum, or obstruction occurring in this bowel, should be sought for before resorting to active remedies. I have known the sudden occurrence of retroversio-uteri give rise to very severe symptoms of obstruction; in another case, attended with much pain, whenever the patient endeavored to force a motion, a hair-pin was found to be firmly fixed by one of its arms, which had penetrated the recto-vaginal septum, whilst the other, remaining free, was pressed upon by the solid contents of the bowel, and inflicted so much pain, as to cause the patient to refrain altogether from straining at stool; the consequence of which was a large accumulation of hardened fæces, which were dislodged by enemata after the foreign body had been removed by the aid of forceps.—*Dublin Medical Press.*

